# Supplementary figures and images for: Clinical impact of ceruloplasmin levels at ANCA-associated vasculitis diagnosis
Source: PLoS One. 2024 Oct 10;19(10):e0311678. doi: 10.1371/journal.pone.0311678 (PMC11466395; doi:10.1371/journal.pone.0311678)

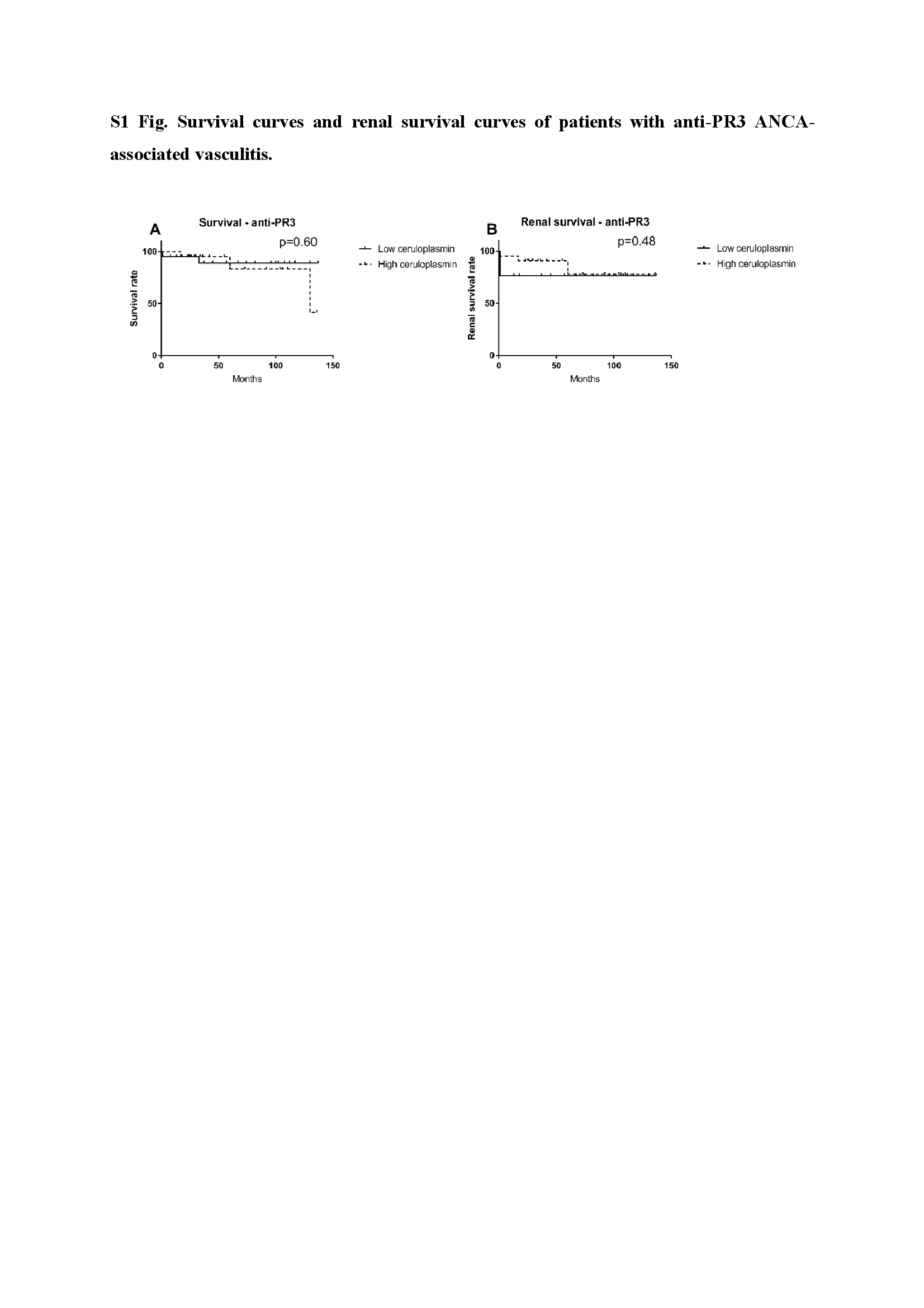

Supplement: S1 Fig — (TIF) [file pone.0311678.s001.tif]

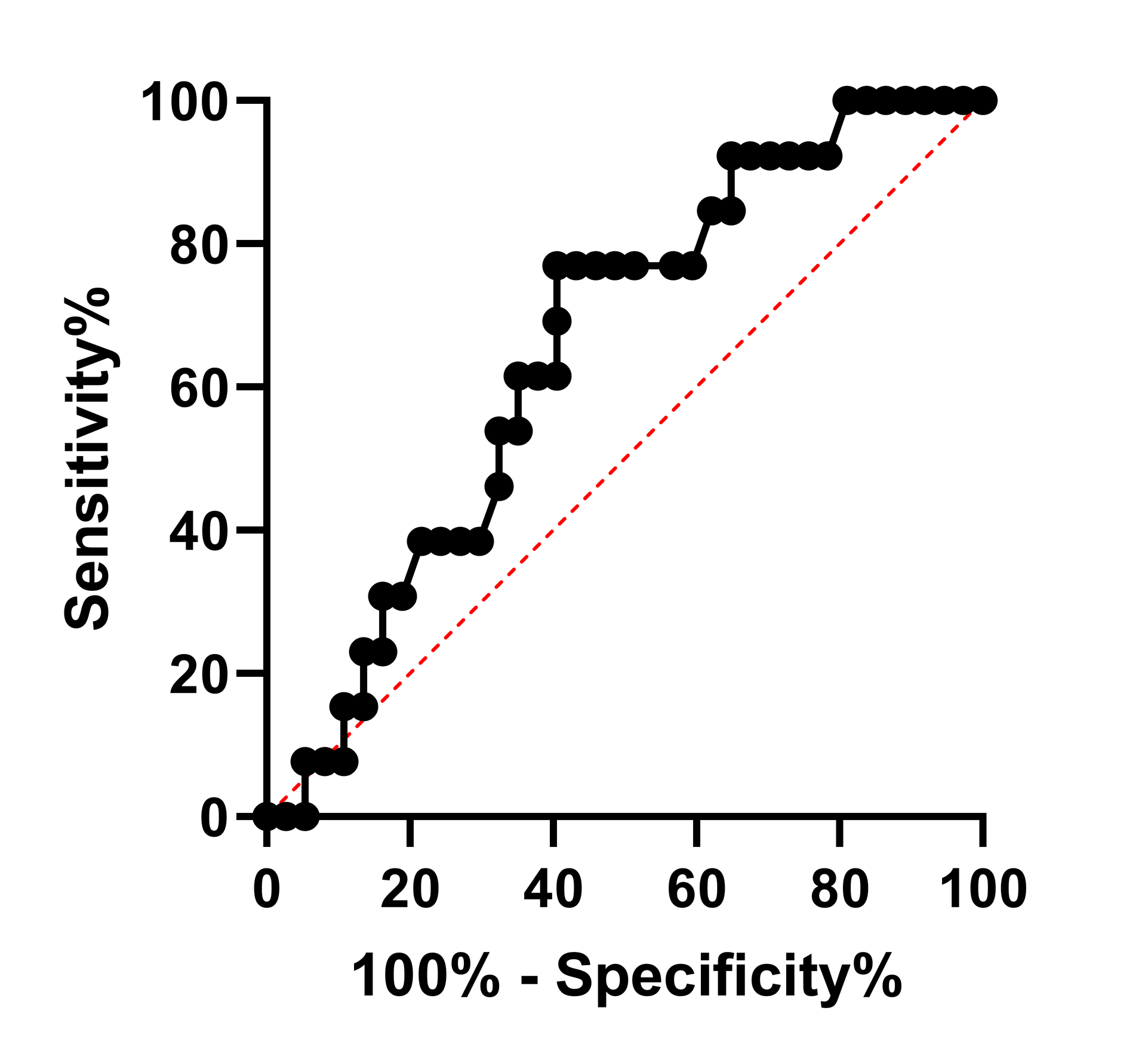

Supplement: S2 Fig — (TIF) [file pone.0311678.s002.tif]

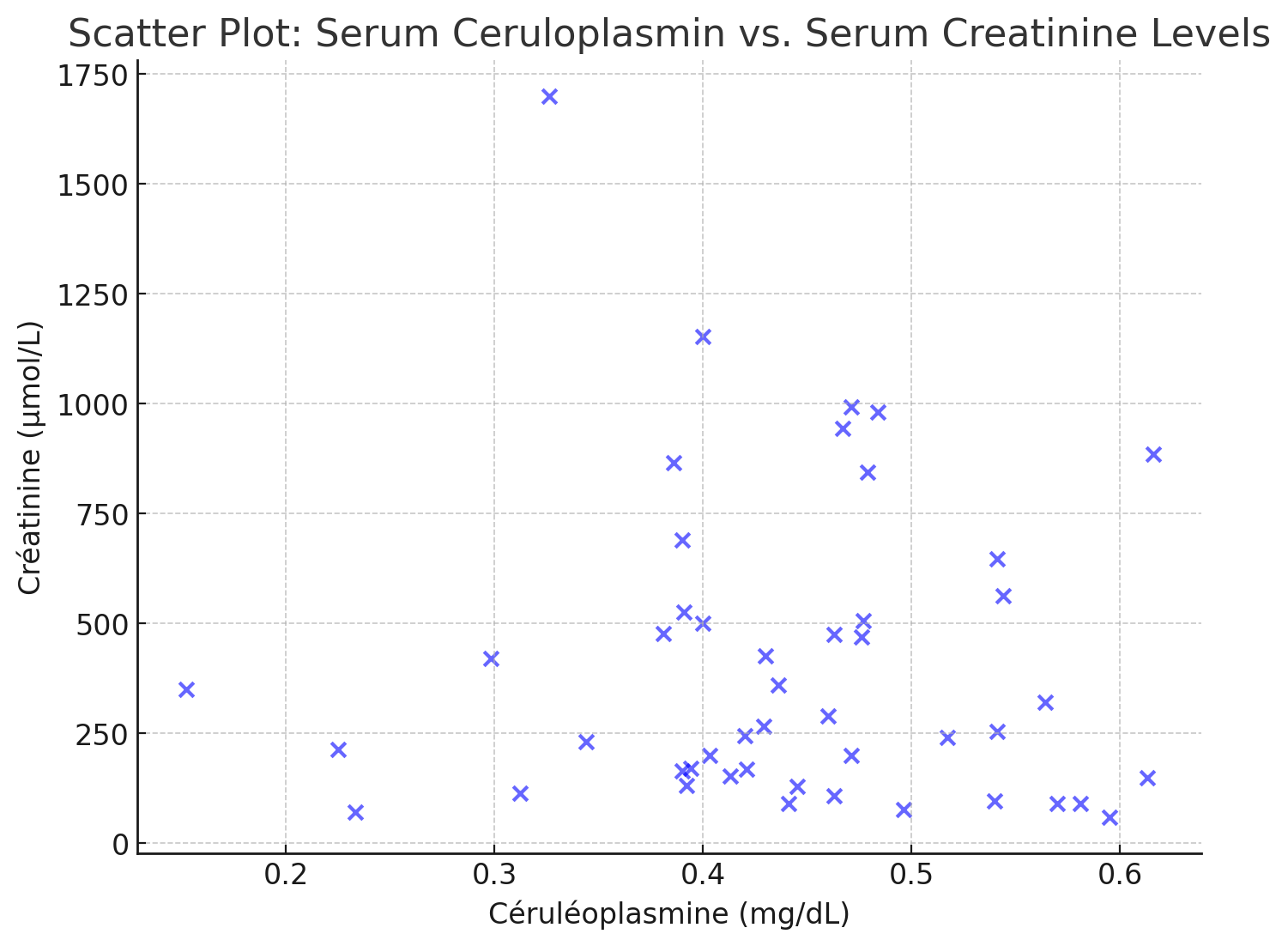

Supplement: S3 Fig — (TIF) [file pone.0311678.s003.tif]
