# Supplementary material for: Clinical impact of ceruloplasmin levels at ANCA-associated vasculitis diagnosis
Source: PLoS One. 2024 Oct 10;19(10):e0311678. doi: 10.1371/journal.pone.0311678 (PMC11466395; doi:10.1371/journal.pone.0311678)
Supplement: S1 Table — Values are given as headcount (%) or median [quartile 1-quartile 3]. GPA: granulomatosis with polyangiitis; MPA: microscopic polyangiitis; BVAS: Birmingham vasculitis activity score; ENT: ear, nose and throat; CRP: C-reactive protein. (DOCX) [file pone.0311678.s004.docx]

**S1 Table. Characteristics of 42 patients with anti-PR3 ANCA-associated vasculitis with ceruloplasmin level available at diagnosis**.

|  | | | | | | | | |  |  |
| --- | --- | --- | --- | --- | --- | --- | --- | --- | --- | --- |
| **Characteristics** | (n=42) | Low ceruloplasmin (n=21) | | High ceruloplasmin (n=21) | | P value | | |  |  |
| **Demographic data** |  |  | |  | |  | | |  |  |
| Age at diagnostic (years) | 63 [50-71] | 63 [47-71] | | 62 [56-69] | | 0.65 | | |  |  |
| Woman | 22 (52) | 11 (52) | | 11 (52) | | 1 | | |  |  |
| **Vasculitis type** |  |  | |  | |  | | |  |  |
| GPA | 41 (98) | 20 (95) | | 21 (100) | | 1 | | | | |
| MPA | 1 (2) | 1 (5) | | 0 (0) | | 1 | | | |  |
| **BVAS** | 19 [14-23] | 20 [16-23] | | 18 [13-22] | | 0.27 | | | | |
| **Characteristics of vasculitis** |  |  | |  | | |  | | | |
| General symptoms | | 35 (83) | 19 (90) | 16 (76) | | 0.41 | | |  |  |
| Dermatological symptoms | 7 (17) | 1 (5) | | 6 (29) | | 0.10 | | |  |  |
| Pulmonary symptoms | 27 (64) | 15 (71) | | 12 (57) | | 0.34 | | |  |  |
| ENT symptoms | 27 (64) | 13 (62) | | 14 (67) | | 0.75 | | |  |  |
| Ophthalmological symptoms | 4 (10) | 1 (5) | | 3 (14) | | 0.61 | | |  |  |
| Abdominal symptoms | 5 (12) | 4 (19) | | 1 (5) | | 0.35 | | |  |  |
| Neurological symptoms | 9 (21) | 3 (14) | | 6 (29) | | 0.46 | | |  |  |
| Cardiological symptoms | 5 (12) | 3 (14) | | 2 (10) | | 1 | | |  |  |
| Renal symptoms | 20 (48) | 11 (52) | | 9 (43) | | 0.54 | | |  |  |
| **Biological data** |  |  | |  | |  | | |  |  |
| Hematuria | 33 (80)  (n=41) | 17 (81) | | 16 (80)  (n=20) | | 1 | | |  |  |
| Proteinuria | 18 (44)  (n=41) | 12 (57) | | 6 (30)  (n=20) | | 0.09 | | |  |  |
| Creatinine level (µmol/L) | 106 [55-383] | 230 [60-577] | | 84 [53-142] | | 0.15 | | |  |  |
| CRP (mg/L) | 150 [63-211]  (n=41) | 169 [79-228]  (n=20) | | 110 [52-185] | | 0.52 | | |  |  |
| **Treatment** |  |  | |  | |  | | |  |  |
| Induction | 38 (90) | 20 (95) | | 18 (86) | | 0.61 | | |  |  |
| Cyclophosphamide | 23 (55) | 15 (72) | | 8 (39) | | 0.03 | | |  |  |
| Rituximab | 20 (48) | 9 (43) | | 11 (53) | | 0.54 | | |  |  |
| Maintenance | 36 (90)  (n=40) | 19 (100)  (n=19) | | 17 (81) | | 0.11 | | |  |  |
| Rituximab | 28 (70) | 14 (74) | | 14 (67) | | 0.63 | | |  |  |
| Azathioprine | 10 (25) | 6 (32) | | 4 (20) | | 0.48 | | |  |  |
| Methotrexate | 3 (8) | 1 (6) | | 2 (10) | | 1 | | |  |  |
| Mycophenolate mofetil | 0 (0) | 0 (0) | | 0 (0) | | 1 | | |  |  |
| Plasma exchanges | 10 (24) | 7 (33) | | 3 (14) | | 0.15 | | |  |  |
| Bolus glucocorticoids | 37 (93) | 19 (100) | | 17 (81) | | 0.11 | | |  |  |
| **Relapses** | 12 (29) | 8 (38) | | 4 (19) | | 0.18 | | |  |  |
| **Deaths** | 5 (12) | 2 (10) | | 3 (14) | | 1 | | |  |  |
| **Chronic end-stage renal disease** | 4 (10) | 3 (14) | | 1 (5) | | 0.61 | | |  |  |
| **Follow-up (months)** | 50 [25-102] | 67 [24-102] | | 36 [26-92] | | 0.57 | | |  |  |

Values are given as headcount (%) or median [quartile 1-quartile 3]

GPA: granulomatosis with polyangiitis; MPA: microscopic polyangiitis; BVAS: Birmingham vasculitis activity score; ENT: ear, nose and throat; CRP: C-reactive protein.
