# Supplementary material for: Clinical impact of ceruloplasmin levels at ANCA-associated vasculitis diagnosis
Source: PLoS One. 2024 Oct 10;19(10):e0311678. doi: 10.1371/journal.pone.0311678 (PMC11466395; doi:10.1371/journal.pone.0311678)
Supplement: S2 Table — Values are given as headcount (%) or median [quartile 1-quartile 3]. GPA: granulomatosis with polyangiitis; MPA: microscopic polyangiitis; PR3: proteinase 3; MPO: myeloperoxidase; BVAS: Birmingham vasculitis activity score; ENT: ear, nose and throat; CRP: C-reactive protein. (DOCX) [file pone.0311678.s005.docx]

**S2 Table. Characteristics of 48 patients with anti-MPO ANCA-associated vasculitis with ceruloplasmin level available at diagnosis, using the 2022 DCVAS criterias for the classification of ANCA-associated vasculitis.**

| **Characteristics** | (n=48) | Low ceruloplasmin (n=24) | | High ceruloplasmin (n=24) | | P value |  |  |
| --- | --- | --- | --- | --- | --- | --- | --- | --- |
| **Demographic data** |  |  | |  | |  |  |  |
| Age at diagnostic (years) | 68 [63-72] | 71 [65-73] | | 67 [63-70] | | 0.18 |  |  |
| Woman | 24 (50) | 12 (50) | | 12 (50) | | 1 |  |  |
| **Vasculitis type** |  |  | |  | |  |  |  |
| GPA | 4 (8) | 2 (8) | | 2 (8) | | 1 | | |
| MPA | 44 (92) | 22 (92) | | 22 (92) | | 1 | |  |
| **BVAS** | 19 [14-22] | 20 [16-22] | | 18 [14-21] | | 0.35 | | |
| **Characteristics of vasculitis** |  |  | |  | |  | |  |
| General symptoms | 39 (81) | 18 (75) | 21 (88) | | 0.47 | |  |  |
| Dermatological symptoms | 6 (13) | 3 (13) | | 3 (13) | | 1 |  |  |
| Pulmonary symptoms | 28 (58) | 13 (54) | | 15 (63) | | 0.56 |  |  |
| ENT symptoms | 11 (23) | 6 (25) | | 5 (21) | | 0.74 |  |  |
| Ophthalmological symptoms | 5 (10) | 3 (13) | | 2 (8) | | 1 |  |  |
| Abdominal symptoms | 1 (2) | 0 (0) | | 1 (4) | | 1 |  |  |
| Neurological symptoms | 11 (23) | 6 (25) | | 5 (21) | | 0.74 |  |  |
| Cardiological symptoms | 1 (2) | 0 (0) | | 1 (4) | | 1 |  |  |
| Renal symptoms | 42 (88) | 22 (92) | | 20 (83) | | 0.67 |  |  |
| **Biological data** |  |  | |  | |  |  |  |
| Hematuria | 40 (83) | 19 (79) | | 21 (88) | | 0.71 |  |  |
| Proteinuria | 34 (71) | 19 (79) | | 15 (63) | | 0.21 |  |  |
| Creatinine level (µmol/L) | 266 [152-516] | 266 [170-489] | | 272 [105-585] | | 0.68 |  |  |
| CRP (mg/L) | 70 [17-148] | 51 [10-107] | | 124 [48-171] | | 0.04 |  |  |
| **Treatment** |  |  | |  | |  |  |  |
| Induction | 43 (90) | 22 (92) | | 21 (88) | | 1 |  |  |
| Cyclophosphamide | 35 (73) | 18 (75) | | 17 (71) | | 0.75 |  |  |
| Rituximab | 8 (17) | 4 (17) | | 4 (17) | | 1 |  |  |
| Maintenance | 38 (79) | 19 (79) | | 19 (79) | | 1 |  |  |
| Rituximab | 15 (32) | 8 (34) | | 7 (30) | | 0.76 |  |  |
| Azathioprine | 21 (44) | 9 (38) | | 12 (50) | | 0.39 |  |  |
| Methotrexate | 1 (3) | 1 (5) | | 0 (0) | | 0.32 |  |  |
| Mycophenolate mofetil | 0 (0) | 0 (0) | | 0 (0) | | 1 |  |  |
| Plasma exchanges | 11 (23) | 5 (21) | | 6 (25) | | 0.74 |  |  |
| Bolus glucocorticoids | 42 (88) | 21 (88) | | 21 (88) | | 1 |  |  |
| Vaccination against *Pneumococcus* | 13 (27) | 4 (17) | | 9 (38) | | 0.20 |  |  |
| Trimethoprim-sulfamethoxazole | 37 (77) | 18 (75) | | 19 (79) | | 0.74 |  |  |
| **Relapses** | 9 (19) | 3 (13) | | 6 (25) | | 0.47 |  |  |
| **Deaths** | 12 (25) | 9 (38) | | 3 (13) | | 0.05 |  |  |
| **Chronic end-stage renal disease** | 10 (21) | 5 (21) | | 5 (21) | | 1 |  |  |
| **Follow-up (months)** | 40 [22-85] | 34 [18-70] | | 49 [23-91] | | 0.14 |  |  |

Values are given as headcount (%) or median [quartile 1-quartile 3].

GPA: granulomatosis with polyangiitis; MPA: microscopic polyangiitis; PR3: proteinase 3; MPO: myeloperoxidase; BVAS: Birmingham vasculitis activity score; ENT: ear, nose and throat; CRP: C-reactive protein.
