# Supplementary material for: Clinical impact of ceruloplasmin levels at ANCA-associated vasculitis diagnosis
Source: PLoS One. 2024 Oct 10;19(10):e0311678. doi: 10.1371/journal.pone.0311678 (PMC11466395; doi:10.1371/journal.pone.0311678)
Supplement: S3 Table — Values are given as headcount (%) or median [quartile 1-quartile 3]. PR3: proteinase 3; MPO: myeloperoxidase; BVAS: Birmingham vasculitis activity score; ENT: ear, nose and throat; CRP: C-reactive protein. (DOCX) [file pone.0311678.s006.docx]

**S3 Table. Characteristics of 45 patients with ANCA-associated vasculitis with a phenotype of granulomatosis with polyangiitis and ceruloplasmin level available at diagnosis, using the 2022 DCVAS criterias for the classification of ANCA-associated vasculitis.**

| **Characteristics** | (n=45) | Low ceruloplasmin (n=22) | | High ceruloplasmin (n=23) | | P value | | |  |  |
| --- | --- | --- | --- | --- | --- | --- | --- | --- | --- | --- |
| **Demographic data** |  |  | |  | |  | | |  |  |
| Age at diagnostic (years) | 64 [51-68] | 64 [48-68] | | 64 [57-69] | | 0.55 | | |  |  |
| Woman | 25 (56) | 12 (55) | | 13 (57) | | 0.89 | | |  |  |
| **ANCA type** |  |  | |  | |  | | |  |  |
| Anti-MPO | 4 (9) | 2 (9) | | 2 (9) | | 1 | | | | |
| Anti-PR3 | 41 (91) | 20 (91) | | 21 (91) | | 1 | | | |  |
| **BVAS** | 20 [14-23] | 20 [16-23] | | 18 [14-23] | | 0.41 | | | | |
| **Characteristics of vasculitis** |  |  | |  | | |  | | | |
| General symptoms | | 37 (82) | 19 (86) | 18 (78) | | 0.70 | | |  |  |
| Dermatological symptoms | 8 (18) | 2 (9) | | 6 (26) | | 0.25 | | |  |  |
| Pulmonary symptoms | 30 (67) | 16 (73) | | 14 (61) | | 0.40 | | |  |  |
| ENT symptoms | 29 (64) | 14 (64) | | 15 (65) | | 0.92 | | |  |  |
| Ophthalmological symptoms | 5 (11) | 2 (9) | | 3 (13) | | 1 | | |  |  |
| Abdominal symptoms | 4 (9) | 3 (14) | | 1 (4) | | 0.35 | | |  |  |
| Neurological symptoms | 9 (20) | 2 (9) | | 7 (30) | | 0.14 | | |  |  |
| Cardiological symptoms | 5 (11) | 3 (14) | | 2 (9) | | 0.67 | | |  |  |
| Renal symptoms | 23 (51) | 12 (55) | | 11 (48) | | 0.66 | | |  |  |
| **Biological data** |  |  | |  | |  | | |  |  |
| Hematuria | 37 (84)  (n=44) | 19 (86) | | 18 (82)  (n=22) | | 1 | | |  |  |
| Proteinuria | 21 (48)  (n=44) | 13 (59) | | 8 (36)  (n=22) | | 0.14 | | |  |  |
| Creatinine level (µmol/L) | 112 [62-422]  (n=44) | 230 [65-577]  (n=21) | | 103 [63-177] | | 0.12 | | |  |  |
| CRP (mg/L) | 150 [51-214]  (n=44) | 168 [17-224]  (n=21) | | 117 [58-195] | | 1 | | |  |  |
| **Treatment** |  |  | |  | |  | | |  |  |
| Induction | 44 (98) | 22 (100) | | 22 (96) | | 1 | | |  |  |
| Cyclophosphamide | 26 (58) | 16 (73) | | 10 (44) | | 0.05 | | |  |  |
| Rituximab | 19 (43) | 8 (37) | | 11 (48) | | 0.44 | | |  |  |
| Maintenance | 38 (88)  (n=43) | 19 (90)  (n=20) | | 19 (83) | | 0.36 | | |  |  |
| Rituximab | 28 (66) | 13 (65) | | 15 (66) | | 0.99 | | |  |  |
| Azathioprine | 12 (28) | 7 (35) | | 5 (22) | | 0.34 | | |  |  |
| Methotrexate | 3 (7) | 1 (5) | | 2 (9) | | 1 | | |  |  |
| Mycophenolate mofetil | 0 (0) | 0 (0) | | 0 (0) | | 1 | | |  |  |
| Plasma exchanges | 12 (27) | 8 (36) | | 4 (17) | | 0.16 | | |  |  |
| Bolus glucocorticoids | 39 (91) | 20 (100) | | 19 (83) | | 0.12 | | |  |  |
| **Relapses** | 14 (31) | 9 (41) | | 5 (22) | | 0.17 | | |  |  |
| **Deaths** | 6 (13) | 3 (14) | | 3 (13) | | 1 | | |  |  |
| **Chronic end-stage renal disease** | 4 (9) | 3 (14) | | 1 (4) | | 0.35 | | |  |  |
| **Follow-up (months)** | 56 [25-102] | 62 [23-102] | | 42 [28-103] | | 1 | | |  |  |

Values are given as headcount (%) or median [quartile 1-quartile 3].

PR3: proteinase 3; MPO: myeloperoxidase; BVAS: Birmingham vasculitis activity score; ENT: ear, nose and throat; CRP: C-reactive protein.
