# Supplementary material for: Clinical impact of ceruloplasmin levels at ANCA-associated vasculitis diagnosis
Source: PLoS One. 2024 Oct 10;19(10):e0311678. doi: 10.1371/journal.pone.0311678 (PMC11466395; doi:10.1371/journal.pone.0311678)
Supplement: S4 Table — Values are given as headcount (%) or median [quartile 1-quartile 3]. PR3: proteinase 3; MPO: myeloperoxidase; BVAS: Birmingham vasculitis activity score; ENT: ear, nose and throat; CRP: C-reactive protein. (DOCX) [file pone.0311678.s007.docx]

**S4 Table. Characteristics of 45 patients with ANCA-associated vasculitis with a phenotype of microscopic polyangiitis and ceruloplasmin level available at diagnosis, using the 2022 DCVAS criterias for the classification of ANCA-associated vasculitis.**

| **Characteristics** | (n=45) | Low ceruloplasmin (n=22) | | High ceruloplasmin (n=23) | | P value | | |  |  |
| --- | --- | --- | --- | --- | --- | --- | --- | --- | --- | --- |
| **Demographic data** |  |  | |  | |  | | |  |  |
| Age at diagnostic (years) | 70 [61-73] | 71 [62-76] | | 67 [62-71] | | 0.22 | | |  |  |
| Woman | 21 (47) | 10 (45) | | 11 (48) | | 0.88 | | |  |  |
| **ANCA type** |  |  | |  | |  | | |  |  |
| Anti-MPO | 44 (98) | 21 (95) | | 23 (100) | | 0.49 | | | | |
| Anti-PR3 | 1 (2) | 1 (5) | | 0 (0) | | 0.49 | | | |  |
| **BVAS** | 18 [14-21] | 19 [15-23] | | 18 [13-21] | | 0.35 | | | | |
| **Characteristics of vasculitis** |  |  | |  | | |  | | | |
| General symptoms | | 37 (82) | 18 (82) | 19 (83) | | 1 | | |  |  |
| Dermatological symptoms | 5 (11) | 2 (9) | | 3 (13) | | 1 | | |  |  |
| Pulmonary symptoms | 25 (56) | 11 (50) | | 14 (61) | | 0.47 | | |  |  |
| ENT symptoms | 9 (20) | 5 (23) | | 4 (17) | | 0.73 | | |  |  |
| Ophthalmological symptoms | 4 (9) | 2 (9) | | 2 (9) | | 1 | | |  |  |
| Abdominal symptoms | 2 (4) | 1 (5) | | 1 (4) | | 1 | | |  |  |
| Neurological symptoms | 11 (24) | 7 (32) | | 4 (17) | | 0.27 | | |  |  |
| Cardiological symptoms | 1 (2) | 0 (0) | | 1 (4) | | 1 | | |  |  |
| Renal symptoms | 39 (87) | 20 (91) | | 19 (83) | | 0.67 | | |  |  |
| **Biological data** |  |  | |  | |  | | |  |  |
| Hematuria | 36 (88)  (n=41) | 17 (81)  (n=21) | | 19 (95)  (n=20) | | 0.35 | | |  |  |
| Proteinuria | 31 (70)  (n=44) | 17 (77) | | 14 (64)  (n=22) | | 0.33 | | |  |  |
| Creatinine level (µmol/L) | 266 [150-526] | 256 [170-495] | | 290 [114-606] | | 0.96 | | |  |  |
| CRP (mg/L) | 80 [19-144]  (n=44) | 63 [12-120] | | 104 [39-167]  (n=22) | | 0.20 | | |  |  |
| **Treatment** |  |  | |  | |  | | |  |  |
| Induction | 40 (89) | 20 (91) | | 20 (87) | | 1 | | |  |  |
| Cyclophosphamide | 32 (72) | 16 (73) | | 16 (70) | | 0.82 | | |  |  |
| Rituximab | 9 (20) | 5 (23) | | 4 (18) | | 0.73 | | |  |  |
| Maintenance | 34 (77)  (n=44) | 16 (76)  (n=21) | | 18 (78) | | 1 | | |  |  |
| Rituximab | 15 (34) | 8 (39) | | 7 (31) | | 0.60 | | |  |  |
| Azathioprine | 19 (44) | 8 (39) | | 11 (48) | | 0.52 | | |  |  |
| Methotrexate | 1 (2) | 1 (5) | | 0 (0) | | 0.48 | | |  |  |
| Mycophenolate mofetil | 1 (2) | 0 (0) | | 1 (4) | |  | | |  |  |
| Plasma exchanges | 9 (20) | 3 (14) | | 6 (26) | | 0.46 | | |  |  |
| Bolus glucocorticoids | 40 (91) | 20 (95) | | 20 (87) | | 0.61 | | |  |  |
| **Relapses** | 7 (16) | 2 (9) | | 5 (22) | | 0.42 | | |  |  |
| **Deaths** | 11 (24) | 8 (36) | | 3 (13) | | 0.07 | | |  |  |
| **Chronic end-stage renal disease** | 10 (22) | 5 (23) | | 5 (22) | | 1 | | |  |  |
| **Follow-up (months)** | 37 [22-82] | 34 [18-75] | | 43 [23-84] | | 0.28 | | |  |  |

Values are given as headcount (%) or median [quartile 1-quartile 3].

PR3: proteinase 3; MPO: myeloperoxidase; BVAS: Birmingham vasculitis activity score; ENT: ear, nose and throat; CRP: C-reactive protein.
