# Supplementary material for: Clinical impact of ceruloplasmin levels at ANCA-associated vasculitis diagnosis
Source: PLoS One. 2024 Oct 10;19(10):e0311678. doi: 10.1371/journal.pone.0311678 (PMC11466395; doi:10.1371/journal.pone.0311678)
Supplement: Attachment — Submitted filename: Response to reviewers.docx [file pone.0311678.s009.docx]

Response to reviewers

Dear Reviewer 1, thank you for your valuable feedback. Please find our responses below.

- To address this first concern, here is the modification we have brough to the introduction “MPO is a key component of neutrophil granules, capable of producing hypochlorous acid (HClO), which contributes to tissue destruction and inhibits α1 anti-trypsin, a plasma inhibitor of PR3. In ANCA-associated vasculitis, MPO is primarily expressed on the surface of activated neutrophils, where anti-MPO antibodies bind to it, triggering the release of reactive oxygen species. This process leads to the formation of neutrophil extracellular traps (NETs), which have cytotoxic properties that cause inflammation and vascular injury.”

We also added the following phrase in the introduction “inadequate inhibition of MPO by low ceruloplasmin levels may contribute to a more severe phenotype in ANCA-associated vasculitis by enhancing oxidative stress and tissue damage through increased MPO activity and interaction with ANCA anti-MPO.”

- We have taken your advice into consideration and modified our results accordingly.
- Regarding the cofounding confounding factors that might influence ceruloplasmin levels, we have added the following details to the manuscript: “Moreover, serum level of ceruloplasmin can be influenced the following parameters: systemic inflammation, hepatic and renal functions (which are frequently disturbed in ANCA-associated vasculitis). Ceruloplasmin serum levels are more often increased during systemic inflammation and impaired renal function, whereas they are decreased in the case of liver failure.”
- We thank you for this highly relevant remark. The low number of deaths in our study makes reliable analysis difficult. The patients who died had not experienced a relapse prior to their death (except for 1 patient), which prevents them from relapsing.

We have made some modifications to the manuscript (in the discussion) in regards to your comments.

“Based on our study's results, decreased ceruloplasmin levels appear to be a marker of poor prognosis in anti-MPO ANCA-associated vasculitis. Although patients with similar BVAS scores did not show a higher relapse rate, the increased number of deaths might be attributed to infectious causes. However, our sample size is too small to confirm this definitively.”

Dear reviewer 2, we thank you for your comments, here are our responses:

- In France, we are not allowed to collect the race of the patients. However, the recruitment is taking place in France, where the majority of GPA cases are found, both nationally and in our center. (<https://link.springer.com/article/10.1186/s41927-024-00385-8>), (<https://pubmed.ncbi.nlm.nih.gov/30805643/>)
- Following your review, we have added the cut off levels of serum ceruloplasmin to discriminate the survival by ROC curve analysis. The ROC curve is added as the S2 Fig.
- There do seem to be polymorphisms that influence ceruloplasmin levels, but these were not investigated in our study.
